# Nature is the best designer: A novel variant of oncolytic reovirus

**DOI:** 10.1016/j.omton.2024.200865

**Published:** 2024-09-05

**Authors:** Raghad Khaleafi, Yotam Bar-On

**Affiliations:** 1 Department of Immunology, Rappaport Faculty of Medicine, Technion-Israel Institute of Technology, Haifa 3525422, Israel

## Main text

Numerous studies have illustrated the potential of using oncolytic viruses for cancer treatment; nonetheless, talimogene laherparepvec (T-VEC) remains the only oncolytic viruses-based therapy that is widely used in the clinic.^1^ The limited success of oncolytic viruses in clinical trials has led to extensive efforts to genetically engineer recombinant variants with improved therapeutic capacities.^2^ In a study published in *Molecular Therapy Oncology*, Song et al. have evaluated the anti-tumor activity of a naturally occurring reovirus variant isolated from persistently infected cancer cells.^3^

This reovirus variant, named RP116, was isolated from persistently infected HT1080 human fibrosarcoma cells and was of special interest due to a premature stop codon at the sigma 1 (σ1) protein. Interestingly, despite lacking the globular head of σ1, which is known to mediate entry of the virus to target cells, RP116 maintained its ability to infect tumor cells. By testing the oncolytic activity of this variant against a panel of cancer cell types, the authors highlighted that the truncated σ1 of RP116 resulted in alterations in the sensitivity of tumor cells to infection; tumor cells with high expression of both sialic acids and integrin β1 were significantly more susceptible to RP116 cytolytic activity, indicating that this variant could be particularly effective against these types of tumors.

Among the main reasons for reoviruses being a promising therapeutic option is their non-pathogenic nature and that, despite constant circulation of the virus in the human population, it is not associated with a particular disease.^2^ On the flip side, prior immunity to a reovirus, particularly preexisting neutralizing antibodies against the σ1 globular head, limits reovirus-based treatment to intratumoral administration.^2^^,^^3^ In accordance with this, the authors demonstrated that RP116 is less sensitive to neutralizing antibodies triggered by the wild-type reovirus. Interestingly, the authors additionally show that a poor neutralizing antibody response is elicited upon administration of RP116, which enables sequential treatment with RP116 followed by administration of a reovirus carrying an intact σ1. This stresses the importance of an in-depth understanding of reovirus interaction with the human immune system, which could facilitate the development of more potent oncolytic treatments. This was also demonstrated by several groups that have engineered recombinant oncolytic viruses capable of boosting the host immune responses for better elimination of the tumor cells.^2^ A prominent example of this is the first US Food and Drug Administration approved oncolytic virotherapy, T-VEC, wherein HSV-1 was engineered to express the granulocyte-macrophage colony-stimulating factor to elicit a stronger immune response.^4^ As for reovirus engineering, the difficulty of manipulating the reovirus genome remains a hurdle in producing enhanced reovirus-based treatments that would elicit a more sustained immune response.^2^ The work of Song et al. offers an alternative approach, where naturally occurring variants with enhanced properties are isolated and studied.^3^

Malignant cells have developed multiple mechanisms to hamper the cytotoxic activity of immune cells.^4^ In this regard, oncolytic virotherapy is no different from other cancer therapeutics, in which drug combination is essential to overcome tumor immunosuppression.^2^ This was also evident in the current study, where infection with RP116 led to increased expression of the immune checkpoint ligand PD-L1 on the infected tumor cells, which, in turn, could significantly impair the activity of cytotoxic T cells by engaging the immune checkpoint receptor PD-1. The possible inhibitory effects of PD-L1 have prompted the authors to evaluate a combination therapy of RP116 and anti-PD-L1-blocking antibodies. Such combination therapy resulted in a striking improvement in tumor elimination in a murine model and in the survival of the mice. Furthermore, this treatment has elicited long-term immune memory that can potentially increase cancer remission.

In light of the various hurdles that are associated with genetic engineering of oncolytic viruses,^2^ this study offers an alternative approach where naturally occurring variants are isolated from tumors and are tested for their therapeutic potential. This is based on the notion that persistent replication of oncolytic viruses in tumor cells likely leads to the emergence of variants with enhanced replication capacity or with other modification that are beneficial for oncolytic activity.^5^ Specifically, the truncated σ1 protein of RP116, which makes it less susceptible to the neutralizing antibodies, warrants further investigation of possible systemic delivery of this variant in clinical trials. The success of such an approach will highly depend on whether prior immunity, directed against antigens outside of the globular head of σ1, will prevent access of RP116 to the tumor microenvironment and will impair the replication of the reovirus in the treated individual.

## Declaration of interests

The authors declare no competing interests.
